# Visual quantification of prostaglandin E_2_ discharge from a single cell

**DOI:** 10.1247/csf.23047

**Published:** 2023-10-07

**Authors:** Tetsuya Watabe, Shinya Yamahira, Michiyuki Matsuda, Kenta Terai

**Affiliations:** 1 Laboratory of Bioimaging and Cell Signaling, Graduate School of Biostudies, Kyoto University, Kyoto 606-8315, Japan; 2 Department of Pathology and Biology of Diseases, Graduate School of Medicine, Kyoto University, Kyoto 606-8315, Japan; 3 Institute for Integrated Cell-Material Sciences, Kyoto University, Kyoto 606-8315, Japan

**Keywords:** prostaglandin E_2_, imaging, intercellular communication, biosensor, quantification

## Abstract

Calcium transients drive cells to discharge prostaglandin E_2_ (PGE_2_). We visualized PGE_2_-induced protein kinase A (PKA) activation and quantitated PGE_2_ secreted from a single cell by combining fluorescence microscopy and a simulation model. For this purpose, we first prepared PGE_2_-producer cells that express either an optogenetic or a chemogenetic calcium channel stimulator: OptoSTIM1 or Gq-DREADD, respectively. Second, we prepared reporter cells expressing the Gs-coupled PGE_2_ reporter EP2 and the PKA biosensor Booster-PKA, which is based on the principle of Förster resonance energy transfer (FRET). Upon the stimulation-induced triggering of calcium transients, a single producer cell discharges PGE_2_ to stimulate PKA in the surrounding reporter cells. Due to the flow of the medium, the PKA-activated area exhibited a comet-like smear when HeLa cells were used. In contrast, radial PKA activation was observed when confluent MDCK cells were used, indicating that PGE_2_ diffusion was restricted to the basolateral space. By fitting the radius of the PKA-activated area to a simulation model based on simple diffusion, we estimated that a single HeLa cell secretes 0.25 fmol PGE_2_ upon a single calcium transient to activate PKA in more than 1000 neighboring cells. This model also predicts that the PGE_2_ discharge rate is comparable to the diffusion rate. Thus, our method quantitatively envisions that a single calcium transient affects more than 1000 neighboring cells via PGE_2_.

## Introduction

Prostaglandin E_2_ (PGE_2_) regulates many homeostatic functions including vascular permeability, immune response, and mucosal integrity (Narumiya, 2007). Although several enzymes are involved in the generation of PGE_2_ from cell membranes, the rate of PGE_2_ synthesis is believed to be regulated primarily by the expression and activity of cyclooxygenases COX1 and COX2 (Kalinski, 2012). Meanwhile, it is also known that the timing of discharge is regulated by cytosolic phospholipase A_2_ (cPLA_2_) (Leslie, 2015; Park *et al.*, 2006). Upon an increase in intracellular calcium, cPLA2 translocates from the cytosol to the Golgi, endoplasmic reticulum, and perinuclear membrane to cleave arachidonic acid out of the membrane phospholipids (Clark *et al.*, 1995; Evans *et al.*, 2001; Hirabayashi *et al.*, 1999). Both COX1 and COX2 use the arachidonic acid to yield prostaglandin H_2_, which is further converted to prostaglandin E_2_ (PGE_2_) by prostaglandin E synthases (Smith and Langenbach, 2001). PGE_2_ generated in the cytosol is impermeable to the cell membrane; therefore, PGE_2_ is secreted to the extracellular space by the prostaglandin transporter PGT (Kanai *et al.*, 1995), the organic anion transporter OAT-PG/Slc22a22 (Shiraya *et al.*, 2010), or by multidrug resistance protein 4 (MRP4) (Reid *et al.*, 2003). The secreted PGE_2_ binds to and activates four G protein-coupled receptors (GPCRs), EP1 to EP4, expressed in neighboring cells (Narumiya, 2007; Regan, 2003). EP2 and EP4 are coupled with Gs. EP1 and EP3 are coupled with Gq and Gi, respectively. Previous studies have quantitated extracellular PGE_2_ by radioimmunoassay or competitive ELISA. Therefore, it was not known how rapidly PGE_2_ is discharged from the cells and to what extent a single PGE_2_-producing cell can modulate nearby cells. Recently, we reported that PGE_2_ discharged from a single Madin-Darby canine kidney (MDCK) cell causes radial spread of PKA activation (RSPA) in neighboring cells and the mouse epidermis (Watabe *et al.*, 2023). We extended this study to quantitate PGE_2_ secretion from a single cell by combining fluorescence microscopy imaging data with a simulation model.

## Results and Discussion

### Visualization of PGE_2_ discharge from a single HeLa cell

**Fig. 1:**
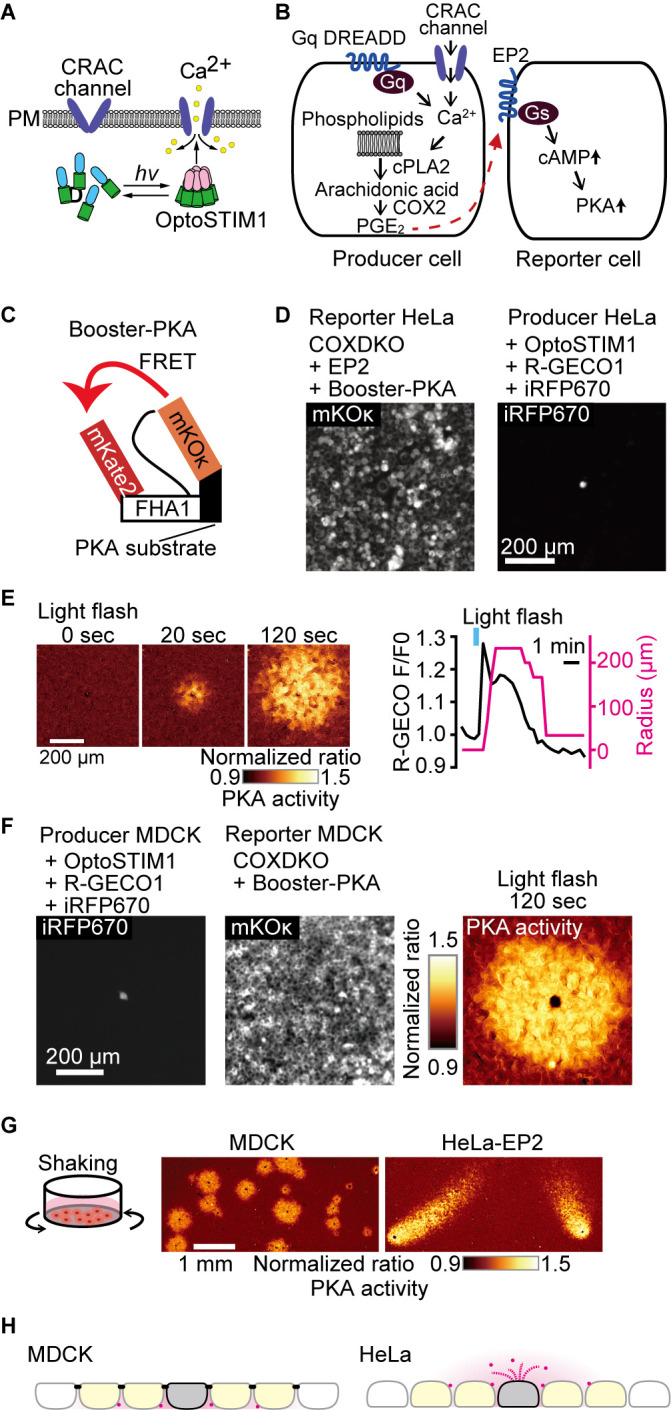
Apical discharge of PGE_2_ from HeLa cells. (A, B) Schematic view of the experimental setup. Either an optogenetic approach to open the CRAC Ca^2+^ channel (A) or a chemogenetic approach to increase cytoplasmic Ca^2+^ via Gq (B) was employed to trigger cPLA2 activation and subsequent PGE_2_ secretion from the producer cell. The secreted PGE_2_ was detected by the reporter cell expressing the Gs-coupled EP2 receptor and Booster-PKA. (C) Structure of Booster-PKA, a FRET biosensor for PKA activity. (D) The Booster-PKA and EP2-expressing HeLa cells, deficient in COX-1 and COX-2 (COX-DKO), were employed as the reporter cells. HeLa cells expressing OptoSTIM1 and R-GECO1 were employed as the producer cells. iRFP670 signal was obtained to identify the producer cells. (E) The producer cells were stimulated by a flashlight during imaging. The FRET ratio, the value of mKate2/mKOκ, in each pixel is shown in pseudocolor as indicated. The time 0 was set as just before blue light irradiation. The F/F0 value of R-GECO1 and the radius of 20% FRET ratio increase were plotted as a function of time. The detection limit for the RSPA radius was 33 μm. (F) The producer and reporter cells derived from MDCK cells were used to observe PKA activation as in 1C, D, and E. (G) Whirlpool effect on the PGE_2_ diffusion. In the left panel, MDCK cells expressing OptoSTIM1 and R-GECO1 were employed as the producer cells. The Booster-PKA-expressing MDCK cells, which were deficient in COX-1 and COX-2 (COX-DKO), were employed as the reporter cells. In the right panel, the producer HeLa cells expressed OptoSTIM1 and R-GECO1, while the COX-DKO HeLa reporter cells expressed Booster-PKA and the exogenous EP2. The producer and reporter cells were mixed and plated in a glass-bottom 24-well plate. Fluorescence and DIC images of multiple positions were acquired every 1 min with an automatically programmable XY stage. The producer cells were stimulated by a flashlight. The mKate2/mKOκ pseudocolor images represented the PKA activity. (H) This scheme represents the directionality of PGE_2_ ejection. The colors represent the following: Magenta, PGE_2_; Gray, producer cells; yellow, PKA-activated reporter cells. The black bars in MDCK cells indicate tight junction.

We recently observed that confluent MDCK cells spontaneously discharge PGE_2_ to cause RSPA (Watabe *et al.*, 2023). To quantitatively analyze this phenomenon, in the present study, we triggered PGE_2_ discharge using calcium transients. For this, we employed either an optogenetic activation of CRAC calcium channel via OptoSTIM1 (Fig. 1A) or a chemogenetic activation of IP_3_ receptor via Gq-DREADD (Fig. 1B). The PGE_2_-mediated PKA activation in the neighboring cells was detected by the FRET biosensor Booster-PKA (Fig. 1C). We attempted to examine whether RSPA can be observed in other cell types such as HeLa cells. In preliminary experiments, however, we failed to observe PKA activation in HeLa cells upon PGE_2_ administration, probably due to the very low expression of EP2 and EP4 in HeLa cells (Liu *et al.*, 2019). Therefore, we developed HeLa reporter cells as follows: First, COX1 and COX2 were knocked out by CRISPR/Cas9 to eliminate PGE_2_ synthesis. Then, EP2 and Booster-PKA were expressed in the COX1/COX2-deficient, hereinafter COX-DKO, HeLa cells to generate the reporter cells (Fig. 1D). The PGE_2_-producer HeLa cells were developed by introducing OptoSTIM1, R-GECO1, and iRFP670. R-GECO1 and iRFP670 were the calcium indicator and the producer cell marker, respectively. The producer cells were co-cultured with the excess of the reporter cells (Fig. 1D). Upon light stimulation, we were able to induce RSPA in HeLa cells (Fig. 1E). This pattern of PKA activation was indistinguishable from that observed with a pair of MDCK producer and reporter cells as reported previously (Fig. 1F) (Watabe *et al.*, 2023). RSPA was not observed in the presence of EP2 and EP4 antagonists, indicating that RSPA is mediated by PGE_2_ (Fig. S1). For the use of the optogenetic tools, cells need to be kept in a dark condition, which is cumbersome for imaging many samples. Thus, we switched to chemogenetic approach, DREADD-Gq, to induce calcium transients. Unexpectedly, however, the PKA-activated cell area exhibited a comet-like smear when we used the HeLa reporter cells, but not MDCK ceporter cells (Fig. 1G, Video 1). We found that this comet-like smear was caused by the whirlpool generated by the stage movement during time-lapse imaging. This unexpected observation revealed that the radial spread of PKA activation in MDCK cells was achieved because the PGE_2_-EP2 signaling was restricted primarily at the basolateral plasma membrane of confluent MDCK cells, which are known to form a mature tight junction (Fig. 1H). Indeed, disruption of adherens junction by E-cadherin knock-out resulted in comet-like smears in MDCK cells (Fig. S2). The prostaglandin transporter PGT localizes at the apical surface of MDCK cells (Endo *et al.*, 2002). Meanwhile, MRP4 and OAT-PG/Slc22a22 localize at the basolateral membrane of MDCK cells and renal proximal tubules (Miah *et al.*, 2016; Shiraya *et al.*, 2010), respectively. Thus, MRP4 and/or OAT-PG/Slc22a22 appear to play the major role in the PGE_2_ discharge from MDCK cells.

### Estimation of the PGE_2_ quantity discharged by a single calcium transient

**Fig. 2:**
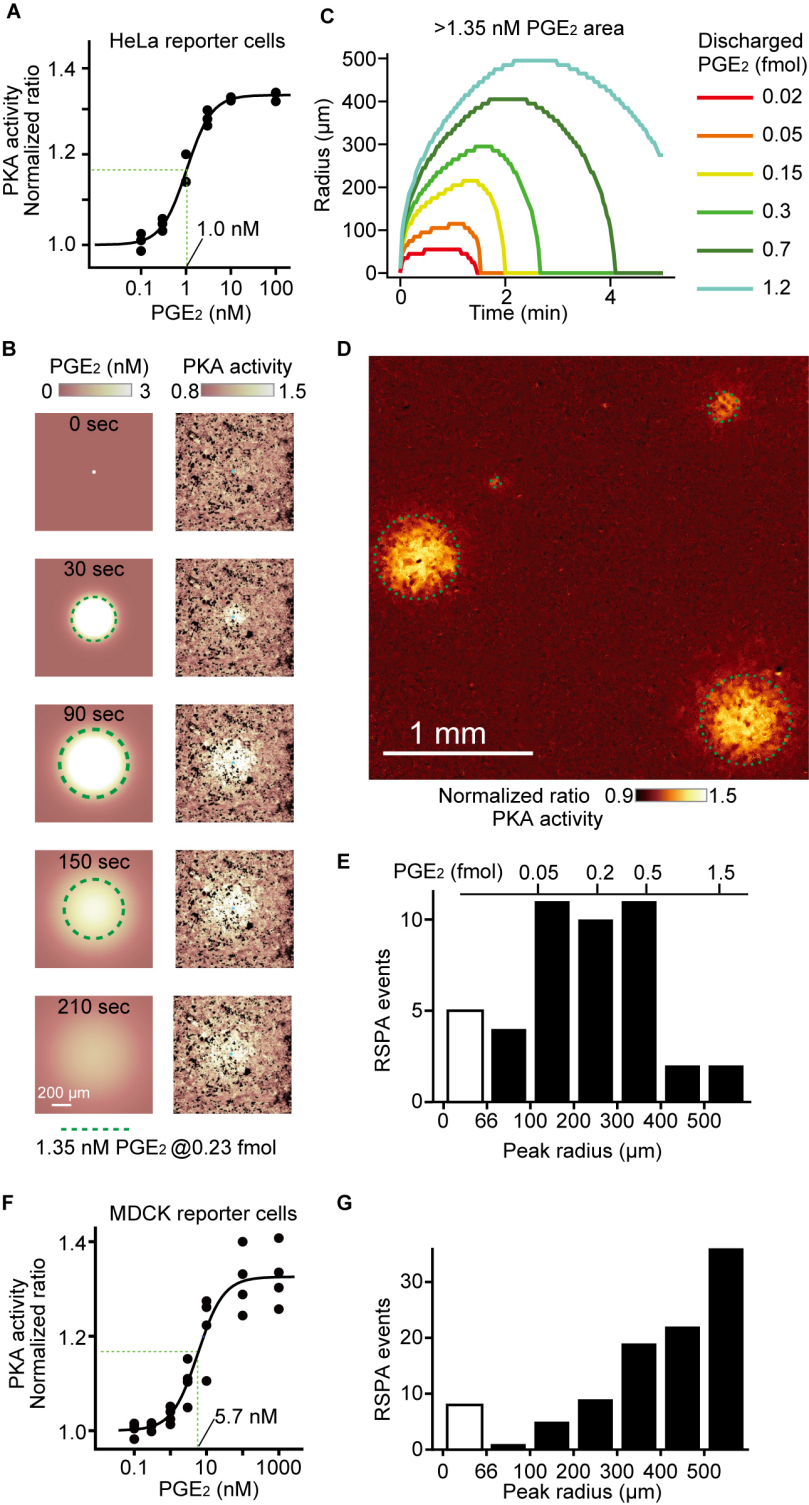
Estimation of the PGE_2_ quantity discharged from a single cell. (A) COX-DKO HeLa cells expressing exogenous EP2 and Booster-PKA were treated with increasing concentrations of PGE_2_. The mKate2/mKOκ ratio representing PKA activity was calculated and plotted against the PGE_2_ concentration. The average intensity of the whole view field of mKate2 or mKOκ, at 20 to 30 min after the addition of PGE_2_, was applied to calculate the mKate2/mKOκ ratio. Three independent experiments were performed. The EC_50_ of Booster PKA activation was determined to be 1.0 nM. (B) A simulation of PGE_2_ diffusion assuming that 0.23 fmol PGE_2_ is ejected from the producer cell (left) and a FRET ratio images of the COX-DKO HeLa cells expressing exogenous EP2 and Booster-PKA (right). Green dotted circles indicate the contours of >1.35 nM PGE_2_ area. (C) The radius of 20% FRET ratio increase, which corresponds to 1.35 nM PGE_2_, was plotted across time for each amount of PGE_2_ discharge. (D) The representative Booster-PKA ratio image 1 minute after blue light flash. The HeLa producer cells expressing OptoSTIM1 and R-GECO1 and the COX-DKO HeLa reporter cells expressing Booster-PKA and the exogenous EP2 were co-cultured and observed as in Fig. 1E. The cells were seeded as follows: HeLa reporter: 10^5^ cells cm^–2^; HeLa producer: 1.0 × 10^2^ cells cm^–2^. Green dotted circles indicate the contours of 20% FRET ratio increase area. (E) The maximum radius of RSPA is represented by the histogram. The detection limit for the RSPA radius was set to 66 μm. The open bar indicates calcium transients without RSPA. From the mode of the peak radius, the PGE_2_ concentration can be estimated as shown at the top of the panel. (F) With COX-DKO MDCK cells expressing Booster-PKA, the dose-response curve was obtained as in (A). The data are reproduced from Fig. 5C of our previous study (Watabe *et al.*, 2023). The EC_50_ of Booster PKA activation was determined to be 5.7 nM. (G) The MDCK producer cells expressing OptoSTIM1 and R-GECO1 and the COX-DKO MDCK reporter cells expressing Booster-PKA were used to prepare the histogram as in (E).

Taking advantage of the free diffusion of PGE_2_ from HeLa cells into the media, we estimated the amount of PGE_2_ secreted after a single calcium transient by combining a simulation model and imaging data. First, the Booster-PKA signal was calibrated by bath application of an increasing amount of PGE_2_ to the HeLa reporter cells (Fig. 2A). From the sigmoidal response curve, the PGE_2_ concentration that gives the half maximal PKA activation was determined to be 1.0 nM (Fig. 2A). Second, we developed a simple 3D diffusion model in the polar coordinates as described in the experimental section. Briefly, we assumed rapid secretion of PGE_2_ from a single producer cell at time zero and adopted the diffusion constant of fluorescein, which has a molecular weight close to that of PGE_2_. The simulation reasonably reproduced the PKA activation observed by the FRET ratio images (Video 2). By using this model, we determined the contour corresponding to 1.35 nM PGE_2_ (Fig. 2B). The radius of the contour at each time point is plotted with various amounts of discharged PGE_2_ (Fig. 2C). The maximum radius during the observation at each concentration was used to generate the calibration bar. Note that, to avoid the whirlpool effect, we performed this experiment without stage movement. During the course of experiments, we noticed that the whirling effect caused by stage movement could be minimized by using 96-well plates, which allowed us to accumulate the RSPA events (Fig. 2D). The histogram analyses indicated that the mean diameter of RSPA was 220 μm (Fig. 2E). From this value, the mean produced PGE_2_ was estimated as 0.25 fmol. Next, we repeated these experiments by using the MDCK reporter cells. The EC_50_ of PGE_2_ was determined to be 5.7 nM, slightly larger than that in HeLa cells (Fig. 2F). The histogram of the size of RSPA shows *ca.* 400 μm as the mean (Fig. 2G). This value is markedly larger than that of HeLa cells, indicating that the simple 3D diffusion model would not be applicable to MDCK cells and that the cell-to-cell junction of the confluent MDCK cells appears to maintain higher concentrations of PGE_2_ at the basolateral surface of confluent MDCK cells.

### Effect of EGF on the PGE_2_ quantity secreted upon a single calcium transient

**Fig. 3:**
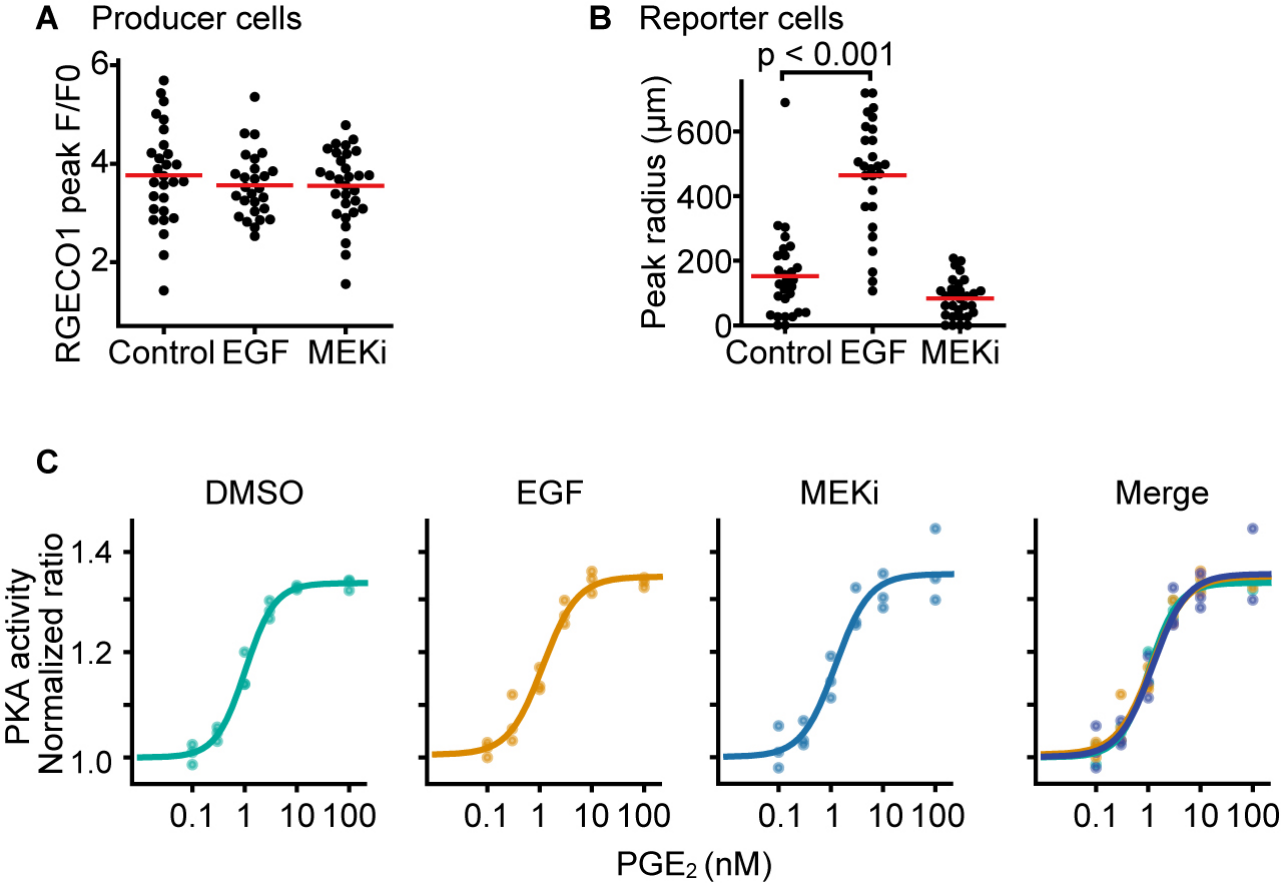
Increased PGE_2_ discharge by EGF stimulation (A, B) The HeLa producer cells expressing OptoSTIM1 and R-GECO1 were mixed with the COX-DKO HeLa reporter cells expressing Booster-PKA and EP2, and plated into 96-well plates. On the next day, cells were incubated for at least 1 h with 0.1% v/v DMSO (control), 50 ng mL^–1^ EGF, or 1 μM PD0325901 (MEKi) before imaging. Images of multiple positions were acquired every 1 min with an automatically programmable XY stage. The producer cells were stimulated by a flashlight. The R-GECO1 fluorescence intensity in the producer cells (A) and the size of RSPA in the reporter cells (B) are shown with the average values (red lines). The detection limit for the RSPA radius was set to 26 μm. Data were analyzed and compared to the control by one-way ANOVA with post hoc Dunnett’s test. (C) COX-DKO HeLa cells expressing exogenous EP2 and Booster-PKA were seeded at 6 × 10^4^ cells cm^–2^, and treated with the indicated concentration of PGE_2_ with 0.1% v/v DMSO, 50 ng mL^–1^ EGF, or 1 μM PD0325901. The mKate2/mKOκ ratio representing PKA activity is plotted against the PGE_2_ concentration. The average intensity of the whole view field of mKate2 or mKOκ, at 20 to 30 min after the addition of PGE_2_, was applied to calculate the mKate2/mKOκ ratio. Data from three independent experiments were fitted and shown for each condition.

We have reported that RSPA in MDCK cells is triggered by calcium transients in cells with high ERK activity (Watabe *et al.*, 2023). To examine whether growth factor signaling affects the amount of PGE_2_ discharge upon a single calcium transient in HeLa cells, the HeLa producer cells expressing OptoSTIM1 with R-GECO1 and the COX-DKO HeLa reporter cells expressing Booster-PKA were mixed and plated into 96-well plates. Cells were pretreated with either EGF or MEK inhibitor for 10 min. EGF pretreatment did not affect the signal intensity of the calcium transients in the producer cells (Fig. 3A), but markedly increased the radius of light-induced RSPA, which reached 500 μm on average, suggesting an ~3-fold increase in the discharged PGE_2_ (Fig. 3B). Meanwhile, the MEK inhibitor modestly reduced the amount of PGE_2_ secretion, suggesting a regulatory role by the ERK MAP kinase. Note that the sensitivity of the reporter cells to PGE_2_ was not affected by either EGF or the MEK inhibitor treatment (Fig. 3C). Therefore, EGF modulates the amount of PGE_2_ secretion after a single calcium transient.

### Quantification of PGE_2_ discharge by ELISA

**Fig. 4:**
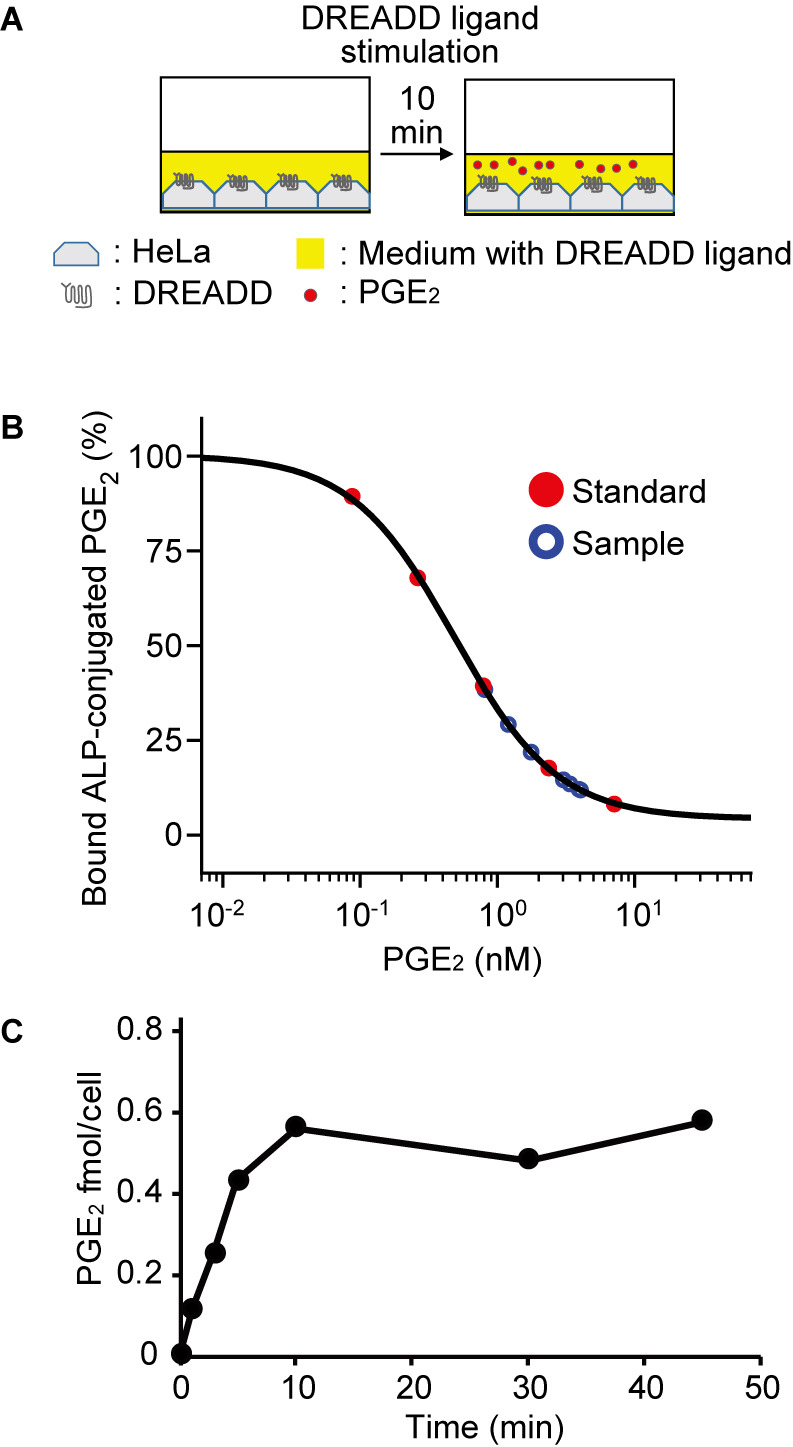
Quantification of PGE_2_ discharge by ELISA (A) Schematic view of the experimental setup. (B, C) Confluent Gq-DREADD-expressing HeLa cells were treated with 1 μM CNO. The supernatant was obtained at the indicated time points and subjected to PGE_2_ quantification with a colorimetric competitive enzyme immunoassay kit. The amounts of secreted PGE_2_ from a single cell were calculated by dividing the total amount of released PGE_2_ by the number of HeLa cells.

In preliminary experiments, we did not find a significant difference in the size of RSPA between the clozapine N-oxide (CNO)-stimulated Gq-DREADD HeLa cells and light-stimulated OptoSTIM1-expressing HeLa cells. Therefore, to achieve the calcium increase uniformly in large number of cells, we adopted Gq-DREADD-expressing HeLa cells as the producer (Fig. 4A). The secreted amount of PGE_2_ was determined by ELISA (Fig. 4B). Upon CNO addition, the amount of PGE_2_ in the medium increased with time, reached a zenith at 10 min, and then remained stable for up to 50 min (Fig. 4C). The PGE_2_ secretion from a single cell was calculated to be 0.5 fmol/cell, which is comparable to the value, 0.25 fmol, estimated by the imaging-based analysis (Fig. 2). This value is also compatible with the previous data obtained by radioimmunoassay. Denning *et al.* reported that calcium ionophore-stimulated 3T3 cells secrete *ca.* 4 nmol PGE_2_/mg protein (Denning *et al.*, 1982). If we assume that the protein weight of a 3T3 cell is 0.1 ng, this value can be transformed to 0.3 fmol/cell. Camacho *et al.* reported *ca.* 300 pmol PGE_2_/10^6^ cells, i.e., 0.3 fmol/cell, in thrombin-stimulated vascular smooth muscle cells (Camacho *et al.*, 2007). Thus, our approach provided fairly reasonable values of stimulation-induced PGE_2_ discharge with additional spatio-temporal information.

### Calcium transient-invoked PKA activation in the neighboring cells

It should be noted that the simulation model (Video 2) reproduced fairly well the PKA activation in the neighboring cells, even though we neglected the export rate of PGE_2_ from the cytosol to the extracellular space. PGE_2_ is exported from the cytosol to the extracellular space in a transporter-dependent manner (Park *et al.*, 2006; Schuster, 2002). Irrespective of whether the transport is passive or active, our observations indicate that the PGE_2_ produced upon the calcium transient is discharged from the producer cell within a few minutes. Owing to this rapid ejection, the secreted PGE_2_ could stimulate PKA in the neighboring HeLa cells at a distance of up to 250 μm from the producer cell. If we assume the area of a single cell to be 200 μm^2^, the PKA-activated area contains *ca.* 1000 cells. Without this fast discharge rate, the PGE_2_ concentration would not reach high enough to trigger the PKA pathway in so many cells. In the case of MDCK cells, which separate the intercellular space from the apical space by tight junctions, the rapid ejection enables PGE_2_ to reach up to 500 μm, which is an area comprising *ca.* 4000 cells.

Calcium transients were originally described as the transient surge of cytoplasmic concentrations in the muscle and neuronal cells (Clapham, 2007). But it is already known that such transient increase in cytoplasmic concentrations is a ubiquitous phenomenon observed in many cell types, including immune cells (Engelke *et al.*, 2007), epithelial cells (Adachi *et al.*, 2016; Murata *et al.*, 2018), and cancer cells (Adachi *et al.*, 2016). Nevertheless, such transient calcium surges have been studied mainly with respect to their effects on intracellular signaling. Our data suggest that a single calcium transient in a single cell could affect a huge number of neighboring cells.

## Experimental Section

### Reagents

ONO-AE3-208, PF-04418948, Prostaglandin E_2_ and the DREADD ligand, clozapine N-oxide, were purchased from Cayman Chemical (Ann Arbor, MI, USA). PD0325901 was purchased from BioVision Inc. (Milpitas, CA, USA). EGF was obtained from Sigma-Aldrich (St. Louis, MO, USA).

### Cell culture

MDCK cells were purchased from the RIKEN BioResource Center (no. RCB0995). HeLa cells were purchased from the Human Science Research Resources Bank. MDCK and HeLa cells were maintained in Dulbecco’s modiﬁed Eagle medium (DMEM; FUJIFILM Wako Pure Chemical Corp., Osaka, Japan) containing 10% fetal bovine serum (Sigma-Aldrich) and 1% v/v penicillin–streptomycin (Nacalai Tesque Inc., Kyoto, Japan).

### Plasmids and primers

Plasmids and primers are described in Table S1A and S1B.

### CRISPR/Cas9-mediated KO cell lines

HeLa cells were transfected with the pX459-derived vectors listed in Table S1A by using 293fectin (Thermo Fisher Scientific, Waltham, MA, USA). 24 h after transfection, cells were treated with the medium containing puromycin. Genomic DNAs were isolated with SimplePrep reagent (TaKaRa Bio, Kusatsu, Japan). PCR was performed using KOD FX neo (Toyobo, Osaka, Japan) for amplification with the primers (Table S1A), followed by DNA sequencing. E-cadherin knock-out MDCK cells were established by using LentiCRISPRv2-derived lentivirus listed in Table S1A. Western blotting was performed to confirm knock-out of E-cadherin (Data not shown).

### Cell lines

The COX-DKO MDCK cells expressing Booster-PKA and the MDCK cells expressing OptoSTIM1 and R-GECO1 were reported previously (Watabe *et al.*, 2023). For the generation of HeLa cells stably expressing Booster-PKA or the other ectopic proteins, a lentiviral or piggyBac transposon system was employed. To establish the reporter HeLa cells, Booster-PKA and EP2 were introduced into COX-DKO HeLa cells via piggyBac transposon system and subjected to single cell cloning. The producer HeLa cells with OptoSTIM1 were established by introducing OptoSTIM1(CRY2clust) and iRFP670-P2Av3-R-GECO1 into the wild-type HeLa cells using piggyBac system and lentivirus, respectively. DREADD-Gq and were established by introducing hM3D-P2Av3-mCherryNLS with YC3.60 or GCaMP6s into the wild-type HeLa cells using piggyBac system, generating the producer HeLa cells with DREADD-Gq. To prepare the lentivirus, pCSIIhyg-iRFP670-P2Av3-R-GECO1, psPAX2, and pCMV-VSV-G-RSV-Rev were co-transfected into Lenti-X 293T cells using polyethylenimine (Polyscience Inc., Warrington, PA, USA). Virus-containing media were collected at 48 or 72 h after transfection, filtered, and applied to target cells with 10 μg mL^–1^ polybrene (Nacalai Tesque Inc.). To introduce ectopic genes using a PiggyBac system, pPB plasmids and pCMV-mPBase(neo-) encoding piggyBac transposase were co-transfected into HeLa cells by using 293Fectin (Thermo Fisher Scientific). Cells were selected with the medium containing the following antibiotics: 10 μg mL^–1^ blasticidin S (FUJIFILM Wako Pure Chemical Corp.), 2.0 μg mL^–1^ puromycin (InvivoGen, San Diego, CA, USA), or 200 μg mL^–1^ hygromycin (FUJIFILM Wako Pure Chemical Corp.). The established cell lines are described in Table S1C.

### Wide-field fluorescence microscopy

Cells were imaged with an ECLIPSE Ti2 inverted microscope (Nikon, Tokyo, Japan). The ECLIPSE Ti2 inverted microscope was equipped with a Plan Fluor 10X or 4X objective, an ORCA Fusion Digital CMOS camera (Hamamatsu Photonics K.K., Hamamatsu, Japan), an X-Cite TURBO LED light source (Excelitas Technologies Corp., Waltham, MA, USA), a Perfect Focus System (Nikon), a TI2-S-SE-E motorized stage (Nikon), and a stage top incubator (Tokai Hit, Fujinomiya, Japan). The following ﬁlters were used for mKOκ and mKate2 imaging: a 555BP10 excitation ﬁlter (Omega Optical, Brattleboro, VT, USA), an FF562Di03 dichroic mirror (Semrock, Rochester, NY, USA), and XF3024 (590DF35) (Omega Optical) and BLP01-633R-25 (Semrock) emission filters for mKOκ and mKate2, respectively; for YC3.60, a 434/32 excitation filter (Nikon), a dichroic mirror 455 (Nikon), and 480/40 and 535-30 emission filters (Nikon) for CFP and YFP, respectively; for GCaMP imaging, a 480/40 (Nikon) excitation filter, a dichroic mirror 455 (Nikon), and a 535/50 emission filter (Nikon); for iRFP670 imaging, an FF01-640/14 excitation filter (Semrock), a dichroic mirror 660 (Nikon), and a 700/75 emission filter (Nikon); for R-GECO1, a 555BP10 excitation filter (Omega Optical), an FF562Di03 dichroic mirror (Semrock), and an XF3024 emission filter (590DF35) (Omega Optical).

### Light-induced calcium transients

HeLa or MDCK cells expressing both R-GECO1-P2A-iRFP670 and OptoSTIM1 (CRY2clust) (Lee *et al.*, 2014) were used as PGE_2_-producer cells (Fig. 1A). To inhibit cell division after seeding, 0.3 and 3 μg mL^–1^ of mitomycin C (FUJIFILM Wako Pure Chemical Corp.) were applied to the HeLa and MDCK producer cells, respectively, for one hour the day before replating. The COX-DKO HeLa cells expressing EP2 and Booster-PKA or the COX-DKO MDCK cells expressing Booster-PKA were used as the PGE_2_ reporter cells. The producer and reporter cells were mixed at a ratio of 1:400 to 1:1,200 and plated on collagen-coated glass-bottom 96-well plates (Matsunami Glass Ind., Osaka, Japan) or 24-well plates (AGC Inc., Tokyo, Japan). After 16 to 32 h of incubation, the culture media were replaced with phenol red-free M199 (Thermo Fisher Scientific) supplemented with 10% fetal bovine serum (Fig. 1C) or M199 supplemented with 0.1% w/v bovine serum albumin (Sigma-Aldrich) (Fig. 3A–C). Cells were imaged by wide-ﬁeld ﬂuorescence microscopy, as described above. During the observation, OptoSTIM1 was activated with 475 nm LED for 200 msec to trigger calcium influx into the cell.

### Quantification of RSPA

The image processing was performed as described previously (Watabe *et al.*, 2023) by using the program code, which is available via GitHub at https://github.com/TetsuyaWatabe-1991/RSPAanalysis. Briefly, images of cells expressing Booster-PKA were subjected to background subtraction and noise reduction. The mKate2/mKOκ ratio image was normalized by a minimum intensity projection along the time axis. The processed images were binarized with a predetermined threshold and processed by morphological opening and closing to refine the RSPA area. Center coordinates and equivalent circle radii were obtained from each RSPA area. If the distance between the center coordinates of RSPA between successive frames was less than 100 μm, they were considered to be the same RSPA.

### Titration of PGE_2_ sensitivity

COX-DKO HeLa cells expressing exogenous EP2 and Booster-PKA or COX-DKO MDCK cells expressing Booster-PKA were seeded on a 96-well glass-bottom plate. Before imaging, the culture media were replaced with phenol red-free M199 supplemented with 0.1% w/v bovine serum albumin (BSA; Sigma-Aldrich) for HeLa or phenol red-free M199 (Thermo Fisher Scientific) supplemented with 10% fetal bovine serum for MDCK. The 96-well plate was imaged by an inverted microscope as described earlier. mKate2 and mKOκ images were obtained in one position for every well at around 5 min intervals. Cells were stimulated with PGE_2_ at the indicated concentrations. The mKate2/mKOκ ratio was quantified from the average intensity of the whole field of view at around 20 to 30 min after the addition of PGE_2_.

### Simulation of RSPA

Diffusion of released PGE_2_ from a single cell was simulated using a diffusion equation. Given that PGE_2_ diffuses symmetrically in the direction of rotation in the culture dish, the diffusion can be represented by the following polar coordinates:


$$\frac{\partial C}{\partial t}=D\left(\frac{\partial^{2}C}{\partial r^{2}}+\frac{1}{r}\frac{\partial C}{\partial r}+\frac{\partial^{2}C}{\partial z^{2}}\right)$$

where *C* is the concentration of PGE_2_, *D* is the diffusion constant, *t* is time, *r* is the radius, and *z* is the height from the bottom of the dish. Because the diffusion constant of PGE_2_ is not reported, we referred to that of fluorescein, which has a molecular weight similar to that of PGE_2_, 436 μm^2^ sec^–1^ in 22.5°C water (Petrášek and Schwille, 2008). For simplicity we set the diffusion constant as 500 μm^2^ sec^–1^. We used the following boundary conditions and an initial condition:


$$\frac{\partial C\left(r,z=0\right)}{\partial z}=0$$

$$\frac{\partial C\left(r=0,z\right)}{\partial r}=0$$

$$C\left(r,z=z_{max}\right)=0$$

$$C\left(r{=r}_{max},z\right)=0$$

$$C\left(t=0\right)=0$$

where *z_max_* is the maximum height of the coordination, and *r_max_* is the maximum radius of the coordination. Numerical analysis for the partial differential equations was performed and visualized in Python using the NumPy and matplotlib libraries. The program code for simulation is available via GitHub at https://github.com/TetsuyaWatabe-1991/RSPAanalysis.

### Analysis of calcium transients

Intracellular Ca^2+^ concentrations in HeLa cells were visualized with a genetically encoded calcium indicator, R-GECO1. For R-GECO1 analysis, F/F0 calcium signals were calculated by assigning the reference F0 using the fluorescence intensity before each blue light flash. To analyze the peak F/F0 value of R-GECO1, F0 was calculated as the minimum projection of fluorescence intensity over the 5 min before each frame. Each cell showing calcium transients was visually checked to exclude the F/F0 elevation caused by flowing debris and misregistration of cells. If two or more adjacent cells showed calcium transients simultaneously, it was counted as a calcium transient.

### Quantification of PGE_2_ by ELISA

HeLa cells expressing Gq-DREADD and YC3.60 were seeded on 12-well plates (2 × 10^5^ cells/well). One day after seeding, the culture media were replaced with FluoroBright DMEM (Thermo Fischer Scientific) supplemented with 10% FBS, 0.1% w/v bovine serum albumin (BSA; Sigma-Aldrich), 1% v/v GlutaMAX (Thermo Fischer Scientific), and penicillin/streptomycin. At 5 h after the medium change, the culture media were replaced with FluoroBright DMEM supplemented with 0.1% w/v BSA, 1% v/v GlutaMAX, penicillin/streptomycin, and 1 μM CNO. After incubation for the designated amount of time, the culture media were collected. The time zero sample was obtained from cells treated without CNO. PGE_2_ concentrations in culture media were quantiﬁed by a colorimetric competitive enzyme immunoassay kit according to the manufacturer’s protocol (ADI-900-001; Enzo Life Sciences, Farmingdale, NY, USA).

### Statistical analysis

All statistical analyses and visualizations were performed in Python using the libraries NumPy, pandas, SciPy, pingouin, matplotlib, and seaborn. No statistical analysis was used to predetermine the sample size.

### Data availability

The data that support the findings of this study are available within the article and its Supplementary Information or from the corresponding author upon reasonable request.

## Author Contributions

Conceptualization, methodology, validation, formal analysis: T.W., K.T.; Investigation: T.W., S.Y. and K.T.; Data curation: T.W., S.Y., and K.T.; Resources: T.W., K.T. and M.M.; Writing – original draft: T.W.; Writing – review & editing: K.T. and M.M.; Supervision: K.T. and M.M.; Project administration: K.T. and M.M.; Funding acquisition: T.W., K.T. and M.M.

## Notes

The authors declare no competing financial interest.

## Supplementary Material

Video 1 Light-induced RSPA in MDCK cells and HeLa cells. The experiments are described in the legend in Fig. 1G. (Left) Producer cells: MDCK cells expressing OptoSTIM1 and R-GECO1. Reporter cells: the Booster-PKA-expressing MDCK cells, deficient in COX-1 and COX-2 (COX-DKO). (Right) Producer cells: HeLa cells expressing OptoSTIM1 and R-GECO1. Reporter cells: the COX-DKO HeLa cells expressing Booster-PKA and the exogenous EP2. The mKate2/mKOκ pseudocolor images representing the PKA activity are shown. Note that cell debris is flowing over the cells in both movies.

Video 2 A simple diffusion model of PGE_2_ and propagation of PKA activation. Video associated with Fig. 2. (Left) a simulation of PGE_2_ diffusion assuming that 0.23 fmol PGE_2_ is ejected from the producer cell. (Right) a FRET ratio video of the COX-DKO HeLa cells expressing exogenous EP2 and Booster-PKA.
