# Supplementary material for: Visual quantification of prostaglandin E2 discharge from a single cell
Source: Cell Struct Funct. 2023 Oct 7;48(2):241–9. doi: 10.1247/csf.23047 (PMC11496778; doi:10.1247/csf.23047)
Supplement: Supplementary file 3 — Supplementary Materials [file csf_48_23047_3.zip › 48_23047_1.pdf]

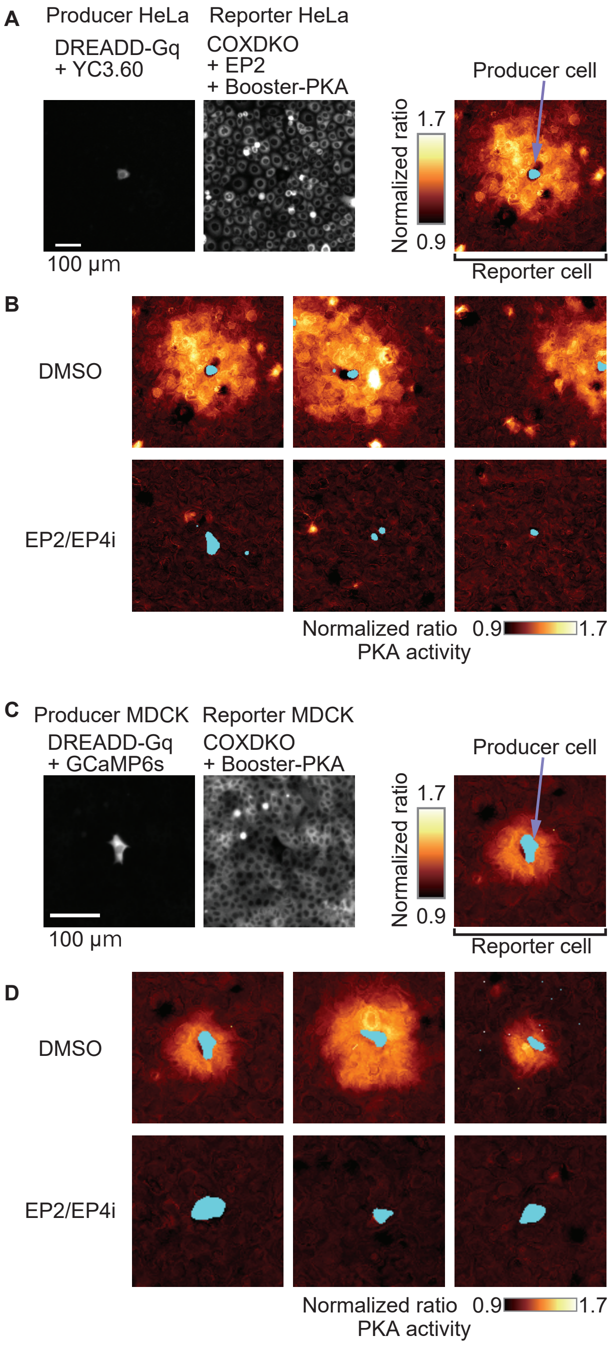


**Figure S1: The Inhibition of RSPA by PGE2 Antagonist.**
**(A)** The experimental setup of RSPA in HeLa cells. HeLa cells expressing DREADD-Gq and calcium biosensor YC3.60 were employed as the producer cells. The Booster-PKA and EP2-expressing HeLa cells, deficient in COX-1 and COX-2 (COX-DKO), were employed as the reporter cells. The FRET ratio in reporter cells was shown in pseudocolor as indicated. The producer cells were represented in cyan color and merged with the FRET ratio image. **(B)** 1 minute after the application of 1 μM DREADD ligand, CNO, in HeLa. Cells were pretreated with DMSO or EP2 and EP4 inhibitors. **(C)** The experimental setup of RSPA in MDCK cells. HeLa cells expressing DREADD-Gq and calcium biosensor GCaMP6s were employed as the producer cells. The Booster-PKA-expressing MDCK cells, deficient in COX-1 and COX-2 (COX-DKO), were employed as the reporter cells. **(D)** 1 minute after the application of 1 μM DREADD ligand, CNO, in MDCK cells. Cells were pretreated with DMSO or EP2 and EP4 inhibitors. Reagents are as follows: DMSO, 0.1% v/v DMSO; EP2i, 10 μM PF-04418948; EP4i, 1 μM ONO-AE3-208.
