# Supplementary material for: Visual quantification of prostaglandin E2 discharge from a single cell
Source: Cell Struct Funct. 2023 Oct 7;48(2):241–9. doi: 10.1247/csf.23047 (PMC11496778; doi:10.1247/csf.23047)
Supplement: Supplementary file 3 — Supplementary Materials [file csf_48_23047_3.zip › 48_23047_2.pdf]

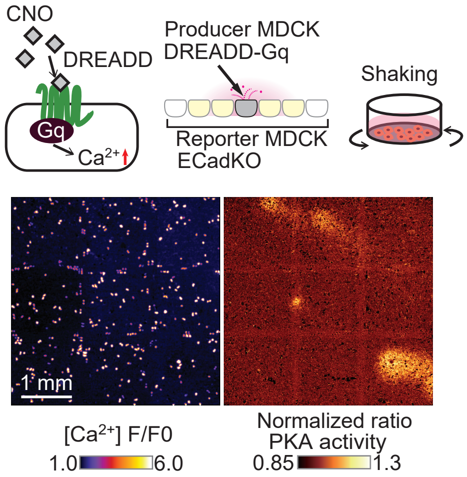

Figure S2: The effect of cell-cell adhesion on RSPA.
2 minutes after the induction of RSPA in MDCK cells with disrupted intercellular adhesion. MDCK cells expressing DREADD-Gq and calcium biosensor, GCaMP6s, were employed as the producer cells. The Booster-PKA-expressing MDCK cells, deficient in E-cadherin, were employed as the reporter cells. The induction of RSPA was performed by the application of 1 μM DREADD ligand, CNO.
